# Differential Effects of C1qa Ablation on Glaucomatous Damage in Two Sexes in DBA/2NNia Mice

**DOI:** 10.1371/journal.pone.0142199

**Published:** 2015-11-06

**Authors:** Ruma Kumari, Konstantin Astafurov, Alina Genis, John Danias

**Affiliations:** 1 Department of Cell Biology, State University of New York (SUNY) Downstate Medical Center, Brooklyn, New York, United States of America; 2 Department of Ophthalmology, State University of New York (SUNY) Downstate Medical Center, Brooklyn, New York, United States of America; 3 State University of New York (SUNY) Eye Institute, Brooklyn, New York, United States of America; Hanson Institute, AUSTRALIA

## Abstract

**Purpose:**

To determine the sex and age-related effects of C1qa ablation on retinal ganglion cell (RGC) and optic nerve (ON) axonal loss in a mouse model of glaucomatous neurodegeneration.

**Methods:**

Congenic C1qa mice were generated in the DBA/2NNia background. Female and male knockout (-/-), heterozygous (+/-), and wild type (+/+) mice were aged up to 14 months and IOPs were recorded in a subset of animals. Retinas of mice from all three groups at 5–6, 9–10 and 11–13 months of age were flat-mounted after retrograde labeling with Fluorogold. Imaged retinas were scored (RGC score) semi-quantitatively on a 10 point scale by two independent observers. A subset of retinas and optic nerves were also used for measurement of total number of RGCs. Semi-thin sections of ON were imaged and graded (ON score) for the amount of axonal damage semi-quantitatively, by two masked observers. Analysis of covariance (ANCOVA) was used for statistical comparisons. Microglial cells in flat-mounted retinas of 5–6 month old C1qa -/- and C1qa +/+ mice were used for assessment of microglial activation utilizing morphological criteria.

**Results:**

Female C1qa -/- mice had significantly higher IOP (p<0.000001, ANOVA) between 8 and 13 months of age compared to C1qa +/+ animals. No differences in IOPs between animals of the three genotypes were observed in males. At 5–6 months of age, there was no difference in RGC or ON scores between the three genotypes in animals of either sex. At 9–10 months of age, female mice didn’t show significant differences in RGC or ON scores between the three genotypes. However, male C1qa -/- and C1qa +/- mice of the same age had better RGC and ON scores (p<0.003 and p<0.05, ANCOVA, for RGC and ON scores, respectively) compared with C1qa +/+ mice. At 11–13 months of age, female C1qa -/- mice had better RGC scores (p<0.006, ANCOVA) compared to C1qa +/+ and C1qa +/- animals. Accordingly, C1qa -/- mice had higher RGC counts (p<0.03, t-test) compared to C1qa +/+ animals. In male mice, there was a tendency for 12 month old C1qa -/- animals to have better RGC scores and higher RGC counts, but this didn't reach statistical significance. ON scores in 11–13 month old animals of either sex were not different between all three genotype. Microglial activation in male 5–6 month old C1qa -/- mice was decreased compared to C1qa +/+ animals; no such effect was seen in females.

**Conclusions:**

Absence of C1qa ameliorates RGC and ON loss in the DBA/2NNia strain, but this effect differs between the two sexes. C1q-mediated RGC damage seems to be more potent than IOP-mediated RGC loss. In contrast, C1qa absence provides axonal protection early on, but this protection cannot overcome the effects of significant IOP elevation.

## Introduction

Glaucoma is a group of neurodegenerative diseases that result in vision reduction through progressive degeneration of the optic nerve and death of Retinal Ganglion Cells (RGCs). Worldwide, it affects more than 60 million people which makes it the second leading cause of blindness [1]. Although elevated intraocular pressure (IOP) and age are the major risk factors that have been associated with the development of glaucoma, the mechanisms through which they influence neuronal loss in this disease are poorly understood. Recently, inappropriate activation of the immune system has been recognized as a potential pathogenetic mechanism of glaucomatous damage. Activation of resident microglia [2, 3] as well as the complement system [4, 5] and Toll-like receptors [6–8] have been implicated in the neurodegeneration process.

The complement system comprises more than 30 proteins present either in soluble or membrane bound form. C1q, the antigen recognition component of the C1 complex, is a 400 kDa protein made up of six copies of three polypeptide chains α, β and γ. Apart from antigen-antibody recognition, C1q is also involved in recognition and removal of apoptotic cells [9]. The removal of dying host cells is essential to maintain tissue homeostasis [10].

Studies have shown that there is upregulation of complement components in various neurodegenerative diseases [11–16]. Specifically C1q has been shown to be upregulated early in the disease in the retina of animal models of glaucoma and in humans with the disease [17]. A large part of our understanding of the cellular mechanisms of this effect comes from the work on DBA/2 mice. DBA/2 is a rodent model of secondary angle closure glaucoma that is useful for studying the molecular mechanisms underlying glaucomatous neurodegeneration [18]. At six to seven months of age these mice start showing anterior chamber abnormalities including iris atrophy, pigment dispersion, anterior synechiae and elevated IOP [19, 20]. By the time animals reach the age of nine months, the majority of mice show these pathological changes. As a result of the increase in IOP, DBA/2 mice develop RGC loss and optic nerve (ON) atrophy starting at 8 months of age for females and at a slightly older age for males [18].

It has previously been reported that C1qa deletion protected DBA/2 mice from glaucomatous neurodegeneration [21]. However, it is still unclear how this effect is mediated. We have initiated the process of breeding C1qa deficient congenic mice in the DBA strain well before the publication of the prior report. In the current report, we provide a detailed characterization of the effect of C1qa absence on RGC and ON pathology in DBA/2 mice. Sex-dependent as well as IOP-dependent differences in the amount of neurodegeneration as well as microglial activation has allowed us a better understanding of the role of C1qa in this process.

## Materials and Methods

This study was carried out in strict accordance with the ARVO Statement for the use of Animals in Ophthalmic and Vision Research and was approved by the Institutional Animal Care and Use Committee (IACUC) of SUNY Downstate Medical Center.

### Animals

Congenic knockout mice were generated by repeated backcrossing of C1qa knockout animals [22] (originally obtained from Dr. Botto and maintained in the C57BL/6 strain) with animals in our DBA/2NNia colony. Backcrossing was performed for 14 generations. Heterozygote offspring from the 14^th^ backcross were then intercrossed to generate C1qa -/-, +/- and +/+ mice. Animals 5–6 months of age (actual ages 4.5–6.99 months), 9–10 months of age (actual ages 8.5–10.49 months) and 11–13 months of age (10.5–13.49 months) were used for the experiments. The animals were kept under a 12-hour light/12-hour dark cycle and fed ad libitum for the duration of the study.

### IOP Measurement in Mice

IOPs of subsets of C1qa -/-, +/- and +/+ animals were measured every two weeks between 4 to 14 months of age by rebound tonometry [23]. All measurements were performed at the same time during the day between (12 and 2pm) to minimize the effect of diurnal IOP variation. The number of eyes used for IOP measurements are listed in Table 1 below.

**Table 1:** Numbers of eyes used for IOP determination at each age group.

	Male (number of eyes)			Female (number of eyes)		
Age(Months)	C1qa +/+	C1qa +/-	C1qa -/-	C1qa +/+	C1qa +/-	C1qa -/-
4	14	28	12	18	8	6
5	40	88	70	78	46	54
6	28	66	60	54	24	42
7	36	40	22	46	24	26
8	40	62	32	42	22	32
9	38	51	22	38	32	32
10	28	36	19	26	42	36
11	38	23	19	26	26	26
12	20	10	5	12	30	24
13	12	8	7	2	20	12
14	14	26	23	0	24	18

### Retrograde RGC labeling and RGC loss assessment

Animals were anesthetized with a mixture of xylazine and ketamine. Their skulls were exposed and holes of approximately 2mm in diameter were drilled 4mm posterior to bregma and 1mm lateral to midline with a dentist’s drill (Dremel, Racine, WI). The superior colliculli were exposed by gentle aspiration of the overlying cerebral cortex. A piece of Gelfoam (Pharmacia and Upjohn, Kalamazoo, MI) soaked in a 5% solution of the neurotracer dye fluorogold (Fluorochrome, Denver, CO) was directly applied to each superior colliculus. Skull openings were then covered with a petrolatum based erythromycin ointment, the overlying skin of skull was sutured and antibiotic ointment was applied externally. After 4 days animals were sacrificed under anesthesia by transcardiac perfusion with 4% PFA. The eyes were enucleated, retinas were removed and flat-mounted. Retrograde fluorogold labeling allows detecting RGCs in the retina that are viable and have intact axon transport function. Although it has been shown that RGCs undergo apoptosis soon after losing axonal transport [24] it is possible that some RGCs may survive for longer periods of time. Therefore, in this manuscript, when referring to RGC death and RGC loss assessed with fluorogold labeling we actually refer to the loss of RGC and loss of RGC axonal transport combined.

Imaging of the fluorogold-labeled retinas was performed with an 10X objective lens with an epi-fluorescent microscope (AxioImager Z1, Zeiss Thornwood NY) equipped with a digital camera (Axiocam HR, Zeiss Thornwood NY) using a wide band ultraviolet excitation filter (appropriate for Fluorogold). The entire retina was imaged using adjacent non-overlapping images in a 9x9 grid pattern. All 81 rectangular frames were merged to generate a single image of each retina. Flat-mounted retinas were scored in a masked fashion by two independent observers as previously described [6]. Semi-quantitative scoring on a 10 point scale was based on the number of remaining RGCs. Retinas with very few or no RGCs visible were given scores of 1 while retinas without apparent RGC loss were given scores of 10. Differences of more than 1 point in scores were resolved by common review and all scores were averaged.

### RGC counts

Automated RGC counts were performed in a subset of the retinas as previously described [25]. Images were initially converted to gray scale and thresholded using Photoshop software (Photoshop, ver. 7.0; Adobe Systems, Inc., San Jose, CA) equipped with extended plugins (FoveaPro, Raindeer Games). ImageTool, ver. 3.0 (University of Texas Health Science Center San Antonio [UTHSCSA], San Antonio, TX) software was then used to obtain a rapid count of all RGCs in each frame.

### Optic nerve damage assessment

Animals were perfused transcardially with 4% paraformaldehyde (PFA) at the time of euthanization and after enucleation, the cranium was opened, and the brain, together with the optic nerves, were removed and immersed in a mixture of 1.2% PFA/0.8% glutaraldehyde for 24 hours at 4°C. The fixative was then washed off, and the optic nerves were osmicated in 2% osmium tetroxide and embedded in epoxy resin (LR White; Electron Microscopy Sciences, Hatfield, PA). Semi-thin sections were cut with an ultramicrotome, stained with *p*-phenylenediamine, mounted, and observed under a microscope equipped with a 63× oil-immersion lens. Nerves were graded in a masked fashion by two independent observers as previously described [6]. Semi quantitative scoring on a five-point scale reflected the amount of axonal damage based on the presence of abnormal or disorganized myelin staining. Nerves with severe axonal degeneration were given a score of 5 while nerves with little or no degeneration were given a score of 1. Differences in scores larger than 1 between the two observers were resolved by common review. Scores of the two observers for each nerve were averaged.

### ON axon counting

Quantitative assessment of optic nerve axon damage was performed in a subset of ONs using the method described by Marina et al [26]. The total nerve area (μm^2^) was determined in images obtained with a 10X objective using Axiovision 4.7 imaging software. Zones of similar damage within the optic nerve were identified, outlined and their respective areas measured. A representative image of each zone was obtained with a 63X objective lens and used for determination of axonal density (axons/ μm^2^) with the Image J software. The total number of axons in the optic nerve was calculated as the sum of the products of axonal density of each zone times its respective area.

### Immunohistochemistry

5–6 month old C1qa -/- and +/+ animals (both males and females) were perfused with 4% PFA, the eyes were enucleated and the retinas were dissected as whole-mounts. They were rinsed with phosphate buffered saline (PBS) and incubated in a blocking buffer (5% donkey serum, 1% BSA, 0.3% Triton-X in 1X PBS) overnight at 4°C. Tissue was then incubated with primary antibody in an antibody buffer (1% donkey serum, 1% BSA, 0.3% Triton-X in 1XPBS) for 24 hours at 4°C. Rabbit anti-Iba1 antibody (WAKO, Richmond, VA) was used at 1:500 dilution. Tissue was washed four times for 30 minutes each with PBS and incubated with an Alexa-Fluor conjugated secondary antibody in the antibody buffer for 24 hours at 4°C. Finally, whole-mounts were washed twice in PBS, incubated in DAPI solution for 20 minutes, washed again twice in PBS, placed on glass slides and cover slipped with the PPD containing mounting medium.

### Morphometric analysis of microglial activation

Immunolabelled retinas were imaged with a laser scanning confocal microscope (Olympus, FV1000) using a 40 × oil-immersion lens (N.A. 1.3) and appropriate laser wavelength. Images were acquired with the resolution of 0.31 μm/pixel at a 30 to 60 μm Z-stack with 2 μm slice thickness. Each quadrant was sampled with the field located 1000 μm from the optic nerve. Microglial cell present in the NFL or in the innermost part of the RGC layer were used for the analysis. The images were saved as 8-bit grayscale tiff files and loaded as an image sequence into the open source Fiji software (http://fiji.sc/Fiji). Tracing of the microglial cells was manually performed in Fiji. The Simple Neurite Tracer plugin was used to manually trace all visible primary, secondary, tertiary, and so on, processes of each cell in the entire Z-stack taking particular care not to include in the trace closely opposed projections from the neighboring cells (as opposed to utilizing a maximum intensity projection image in which distinction between processes from adjacent cells is usually impossible). Traces were then saved as a separate file and converted to a binary image. Following that, the cell trace position was adjusted so that the center of each traced cell nucleus was placed exactly at the center of the image.

Sholl analysis of the resultant binary files was performed in Fiji software using Sholl Analysis tool with the following settings: Sholl method = Intersections, starting radius = 2μm, ending radius = 150μm, radius step size = 1μm, samples per radius = 1. A number of parameters including the average and maximum number of intersections from the soma was recorded and analyzed.

### Statistical analysis

IOP values of the animals of each sex were analyzed separately. IOP of animals of the three genotypes were compared using analysis of covariance (ANCOVA) with age as covariate.

Optic nerve and retinal scores were also analyzed separately for the two sexes in each age group (5–6, 9–10 and 11–13 months of age) using ANCOVA with age as covariate. RGC counts were compared between C1qa -/- and +/+ congenic animals of the 9–10 and 11–13 age groups separately for each sex using t-test. Comparison of the number of animals at each age group for the level of severity of glaucomatous damage was performed by chi-square testing. A p value of <0.05 was considered statistically significant. All statistical analysis was performed with the NCSS statistical software.

## Results

### Female C1qa -/- have higher IOP than C1qa +/+

**Fig 1:**
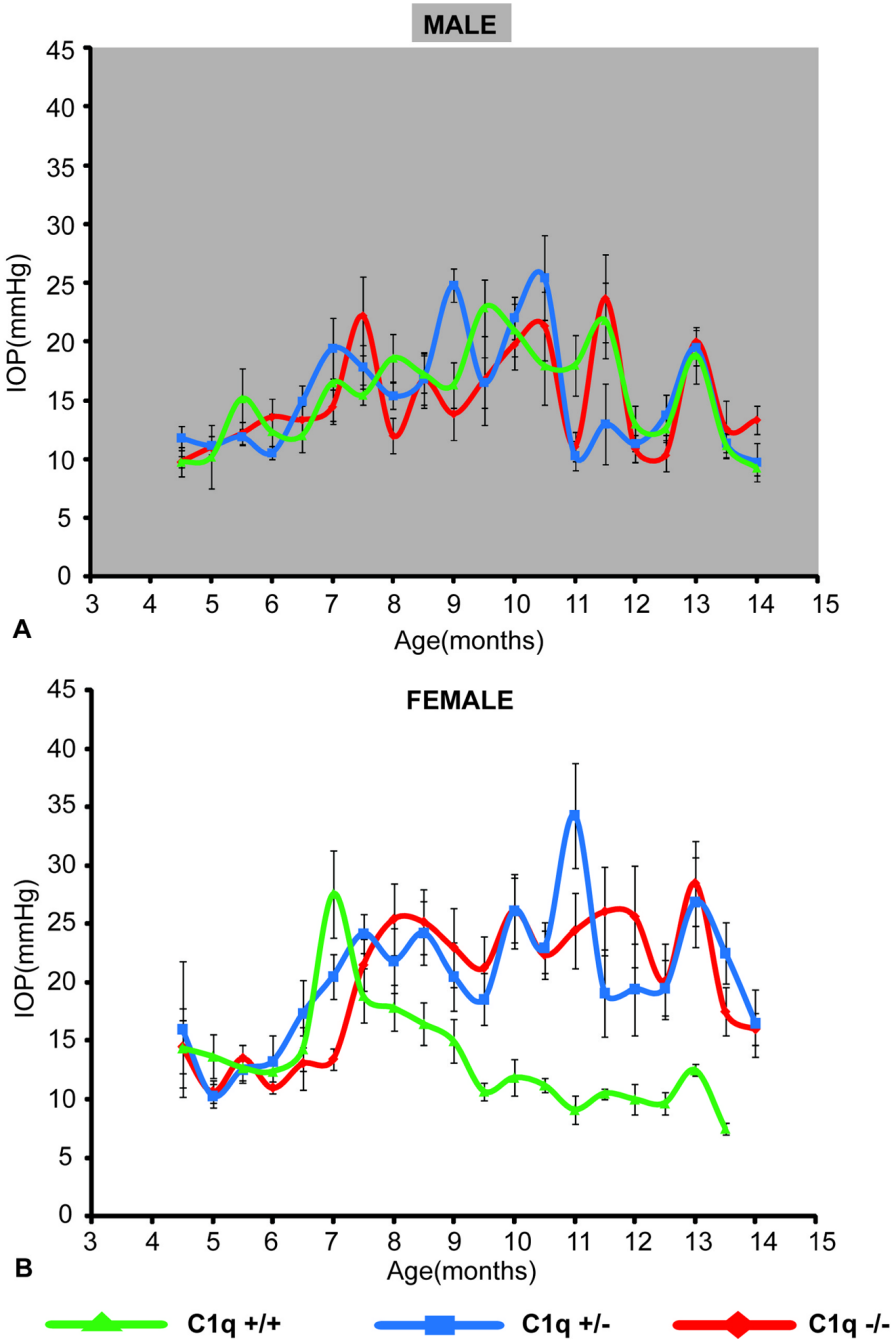
IOPs of a subset (see Table 1) of male (A) and female (B) mice used in this study. Average IOP of male mice was not significantly different between the 3 genotypes (*p>0*.*09*, ANCOVA). In contrast, female C1qa -/- and C1qa +/- mice had higher IOPs than their C1qa +/+ littermates (*p<0*.*000001*, ANCOVA). Error bars represent standard errors of mean at each age.

Both male and female mice showed an increase in IOP from 6 months onwards. IOP elevation was sustained in some animals up to 12 months of age. In male mice, no significant differences in IOP were detected between the three genotypes (*p>0*.*098*, ANCOVA, Fig 1A). In contrast, in female animals, C1qa -/- and C1qa +/- mice had significantly higher IOP compared to C1qa +/+ from ~8 to 13 months of age (*p<0*.*05*, post-hoc Fisher's LSD, *p<0*.*000001*, ANCOVA, Fig 1B).

### Semi-quantitative scoring correlates well with counting

**Fig 2:**
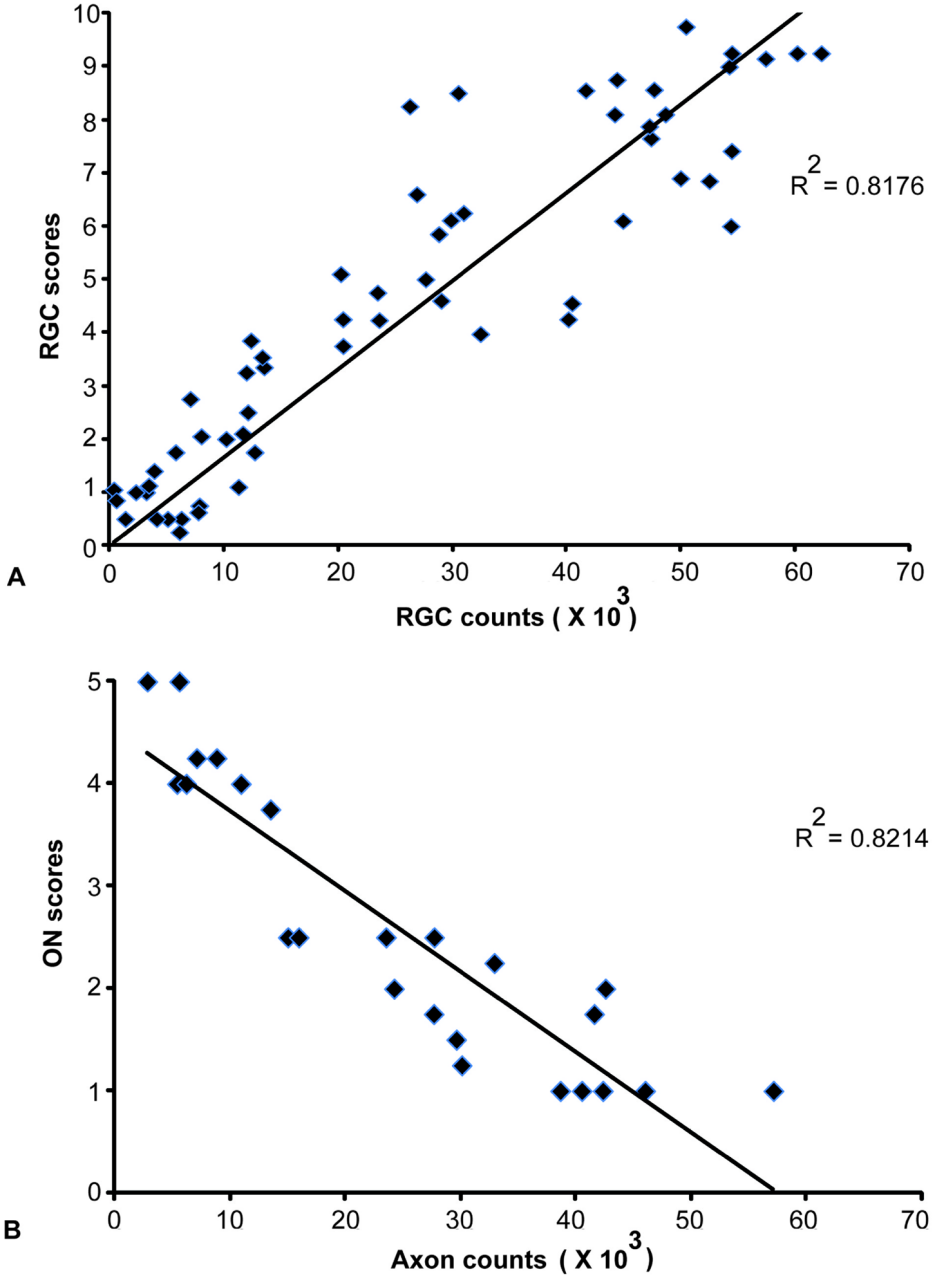
Correlation between semi-quantitative scoring and counting of RGCs (A) and ON axons (B). Linear regression coefficients (*R* ^*2*^) are indicated on the graphs.

Although the methods of semi-quantitative scoring of retinas and optic nerves have been previously validated, to ensure the accuracy of these grading methods in this particular cohort of animals, we counted the number of RGCs and ON axons in a subset of eyes using established methodology (see methods section). In the cohort of animals reported here, both the RGC scoring and ON scoring showed good correlation with the total RGC counts and total ON axon counts respectively (*R* ^*2*^ *= 0*.*82* for correlation between RGC scores and counts (N = 59 eyes) and *R* ^*2*^ *= 0*.*82* for ON scores and axonal counts (N = 24)) (Fig 2A and 2B).

### C1qa absence attenuates RGC death

In order to determine the effect of C1qa deletion on RGC and axonal protection animals were divided in three age groups: 5–6 months of age, 9–10 months of age, and 11–13 months of age.

**Fig 3:**
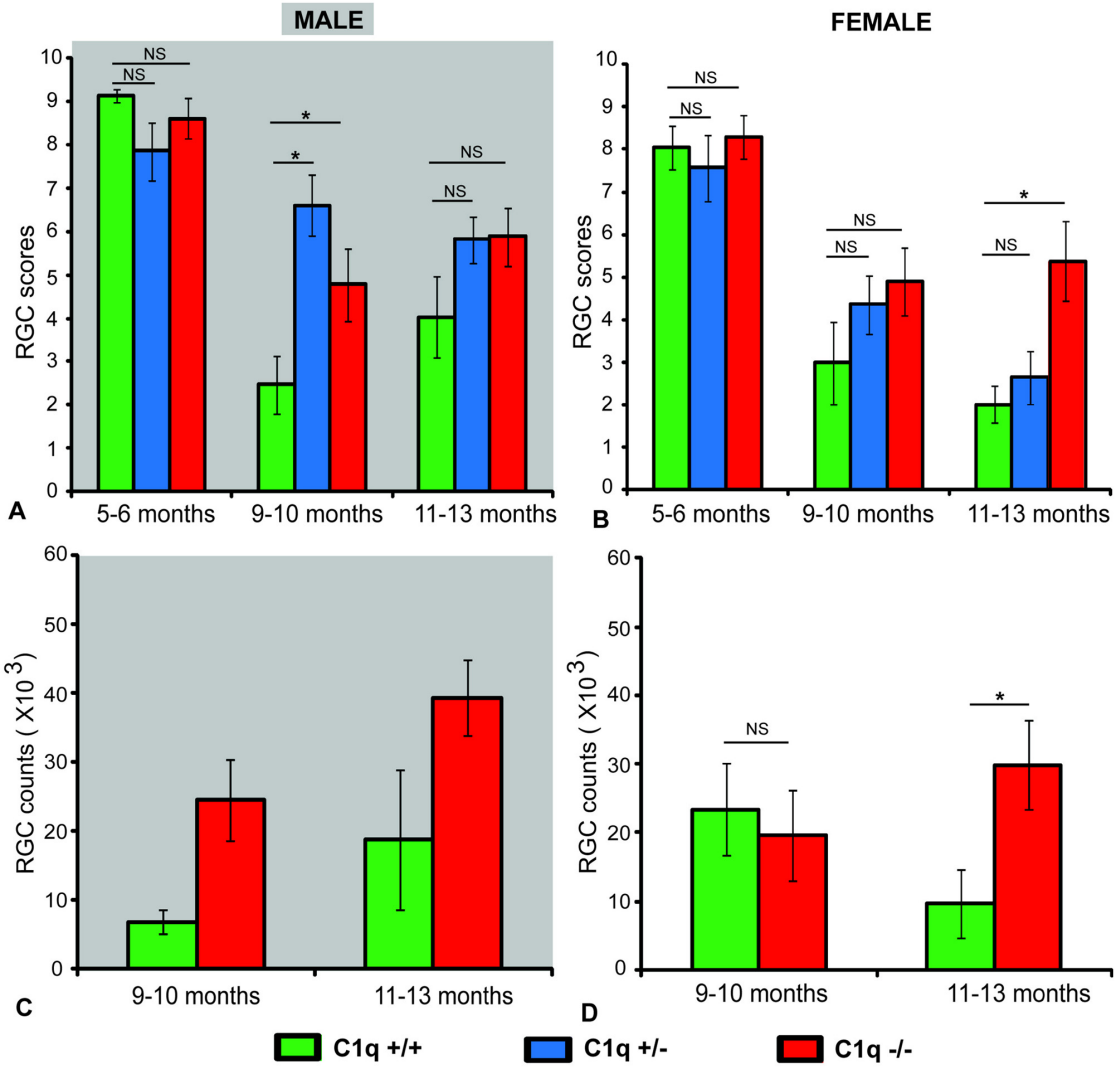
Mean (± SEM) semi-quantitative RGC scores of male (A) and female (B) congenic C1qa DBA/2 mice and RGC counts of a subset of the eyes of male (C) and female (D) animals. Animals are grouped in three age groups: 5–6 months of age, 9–10 months of age and 11–13 months of age. Sample sizes: 18 C1q +/+, 19 C1q +/-, 22 C1q -/- at 5–6 months, 14 C1q +/+, 18 C1q +/-, 18 C1q -/- at 9–10 months and 13 C1q +/+, 34 C1q +/-, 22 C1q -/- at 11–13 months in male mice. 14 C1q +/+, 16 C1q +/-, 21 C1q -/- at 5–6 months, 11 C1q +/+, 21 C1q +/-, 16 C1q -/- at 9–10 months, 11 C1q +/+, 21 C1q +/-, 16 C1q -/- at 11–13 months in female mice. Only eyes from mice in the 9–10 and 11–13 age groups were subjected to RGC counting. Sample sizes: 9 C1q +/+, 14 C1q-/- at 9–10 months, 6 C1q +/+, 11 C1q -/- at 11–13 months in males, 7 C1q +/+, 10 C1q -/- at 9–10 months, 5 C1q +/+, 14 C1q -/- at 11–13 months in females. Statistically significant differences in post-hoc comparisons are indicated by a (*). NS: no statistical significance.

**Fig 4:**
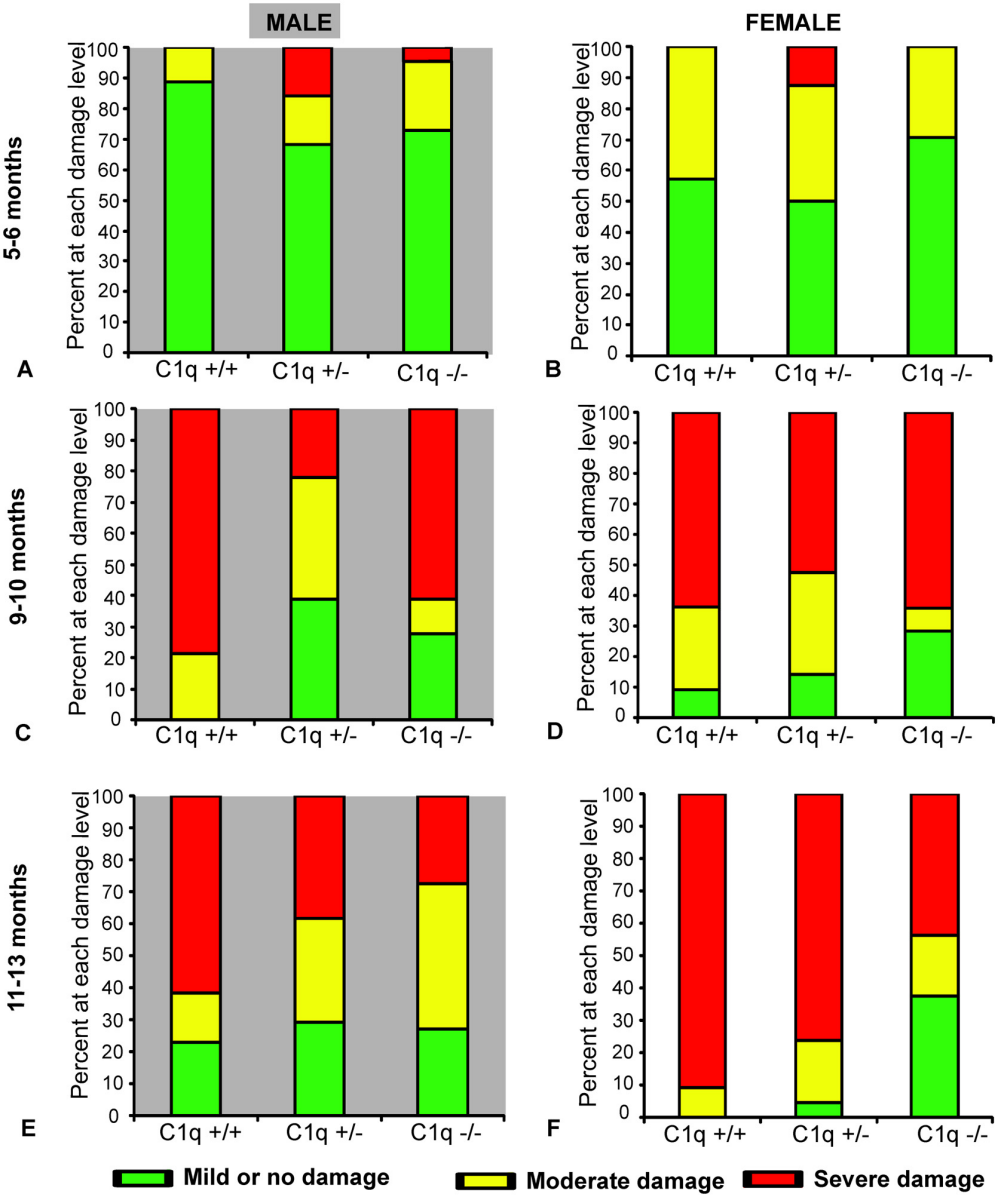
Proportion of eyes from male (A, C, E) and female (B, D, F) congenic C1qa DBA/2 mice with various levels of RGC loss at 5–6 (A, B), 9–10 (C, D) and 11–13 (E, F) months of age. RGC loss was categorized as mild or no damage for eyes with RGC scores >8, moderate for eyes with scores >4 and ≤8 and severe for eyes with scores ≤4.

In male animals 5–6 months of age, RGC scores were not significantly different between C1qa +/+, C1qa +/-, and C1qa -/- genotypes (*p>0*.*14*, ANCOVA, Fig 3A). The proportion of male animals with mild (RGC scores >8), moderate (RGC scores >4 and ≤8) or severe (RGC scores ≤ 4) damage was also not significantly different between the three genotypes at 5–6 months of age (*p>0*.*28*, Fisher exact test, Fig 4A). At 9–10 months of age RGC scores of C1qa -/- and C1qa +/- animals were significantly higher compared to those of C1qa +/+ animals (*p<0*.*05*, post-hoc Fisher's LSD, *p<0*.*003*, ANCOVA, Fig 3A). 100% (14 of 14) eyes from C1qa +/+ mice in this age group were either severely or moderately damaged while severe or moderate RGC loss was present in only 61% (11 of 18) eyes of C1qa +/- and 72% (13 of 18 eyes) of C1qa -/- mice (*p<0*.*01*, Fisher exact test, Fig 4C). At 11–13 months of age, C1qa -/- and +/- animals had only marginally higher RGC scores (but differences were not statistically significant) compared to C1qa +/- and +/+ animals (*p>0*.*14*, ANCOVA, Fig 3A). Similarly, the proportion of animals with mild, moderate, or severe damage was also not significantly different between the three genotypes at this age group (*p>0*.*3*, Fisher exact test, Fig 4E).

In female mice at 5–6 months of age, there was no significant difference (*p>0*.*63*, ANCOVA) in mean scores of RGC between C1qa +/+, C1qa +/-, and C1qa -/- animals (Fig 3B). The majority of eyes from animals of all three genotypes had mild or no damage with no significant differences in the proportions of various degrees of damage detected between genotypes (*p>0*.*73*, Fischer exact test, Fig 4B). At 9–10 months of age, there was also no significant difference (*p>0*.*23*, ANCOVA) of mean RGCs scores in all three genotypes (Fig 3B). The proportion of retinas at each damage level was also not significantly different between the three genotypes (*p>0*.*36*, Fischer exact test, Fig 4D). At 11–13 months of age there was a significantly higher mean RGC score (*p<0*.*05*, post-hoc Fisher's LSD, *p<0*.*006*, ANCOVA) in C1qa -/- mice compared to C1qa +/+, and C1qa +/- mice (Fig 3B). In C1qa +/+ mice 91% of eyes (10 of 11) had severely damaged retina and 9% (1 of 11) had moderately damaged retina while only 44% of C1qa -/- mouse eyes (7 of 16) had severe damage and 19% (3 of 16) had moderate RGC damage (*p<0*.*021*, Fischer exact test, Fig 4F).

Comparison of RGC counts performed in a subset of eyes from C1qa +/+ and C1qa -/- animals revealed similar results. In males, the mean number of RGCs in eyes of C1qa -/- animals were significantly higher from those in eyes of C1qa +/+ animals at 9–10 months, but only marginally (and not statistically significant) different at 11–13 months of age (*p<0*.*03*, *p>0*.*11* respectively, t-test, Fig 3C). Despite the apparent lack of statistically significant difference among mean RGC counts at 11–13 months of age (Fig 3C), a higher proportion of eyes from male C1qa +/+ had extreme RGC loss (less than 10,000 RGCs) (*p<0*.*013*, Fisher exact test). In females, at 9–10 months of age, no significant difference in mean RGC numbers between eyes from C1qa +/+ and C1qa -/- animals was observed (*p>0*.*69*, t test), whereas mean RGC numbers were significantly different among eyes from animals of the same groups at 11–13 months of age (*p<0*.*03*, t-test, Fig 3D).

### C1qa absence retards, but does not prevent axonal damage in male animals

**Fig 5:**
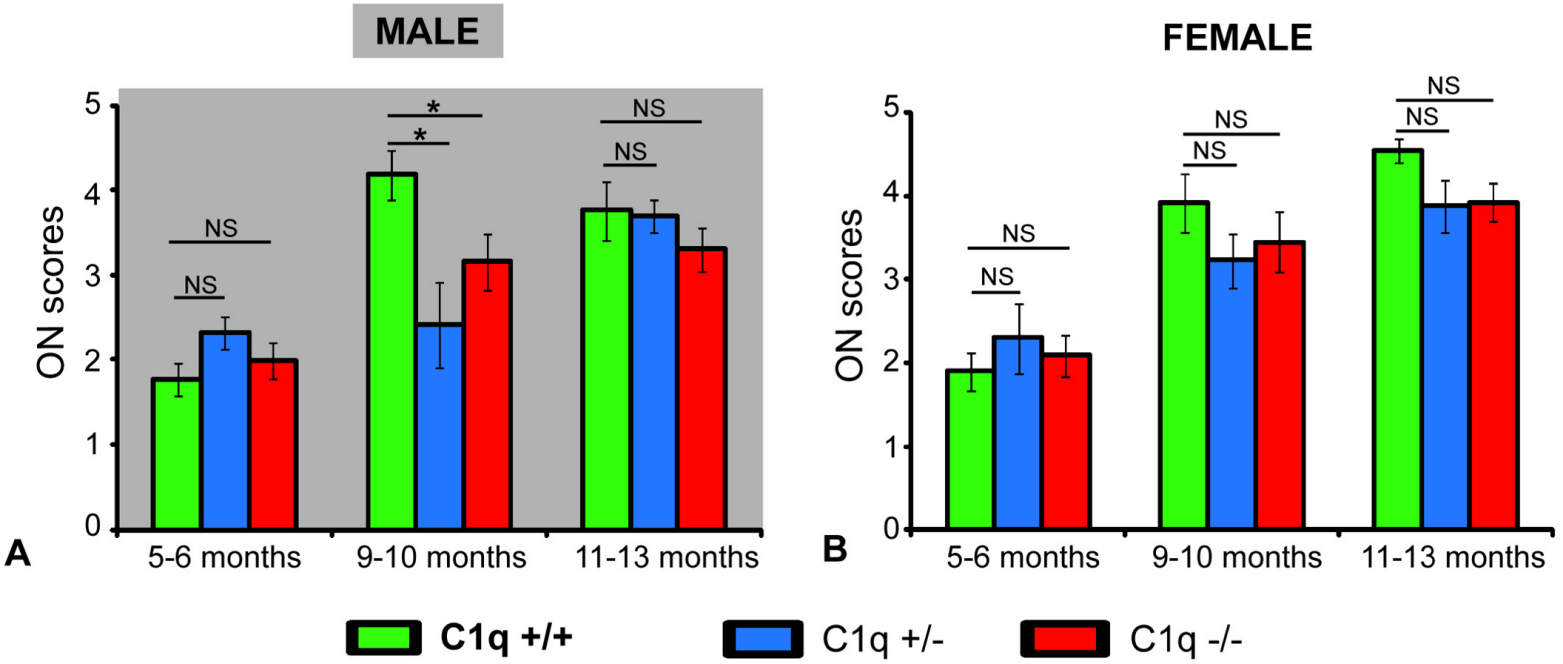
Mean (± SEM) semi-quantitative ON scores of male (A) and female (B) congenic C1qa DBA/2 mice. Animals are grouped in three age groups: 5–6 months of age, 9–10 months of age and 11–13 months of age. Sample sizes: 22 C1q +/+, 32 C1q +/-, 12 C1q -/- at 5–6 months, 11 C1q +/+, 6 C1q +/-, 16 C1q -/- at 9–10 months and 11 C1q +/+, 36 C1q +/-, 18 C1q -/- at 11–13 months in male mice. 12 C1q +/+, 13 C1q +/-, 19 C1q -/- at 5–6 months, 11 C1q +/+, 17 C1q +/-, 13 C1q -/- at 9–10 months, 27 C1q +/+, 16 C1q +/-, 25 C1q -/- at 11–13 months in female mice. Statistically significant differences in post-hoc comparisons are indicated by a (*). NS: no statistical significance.

**Fig 6:**
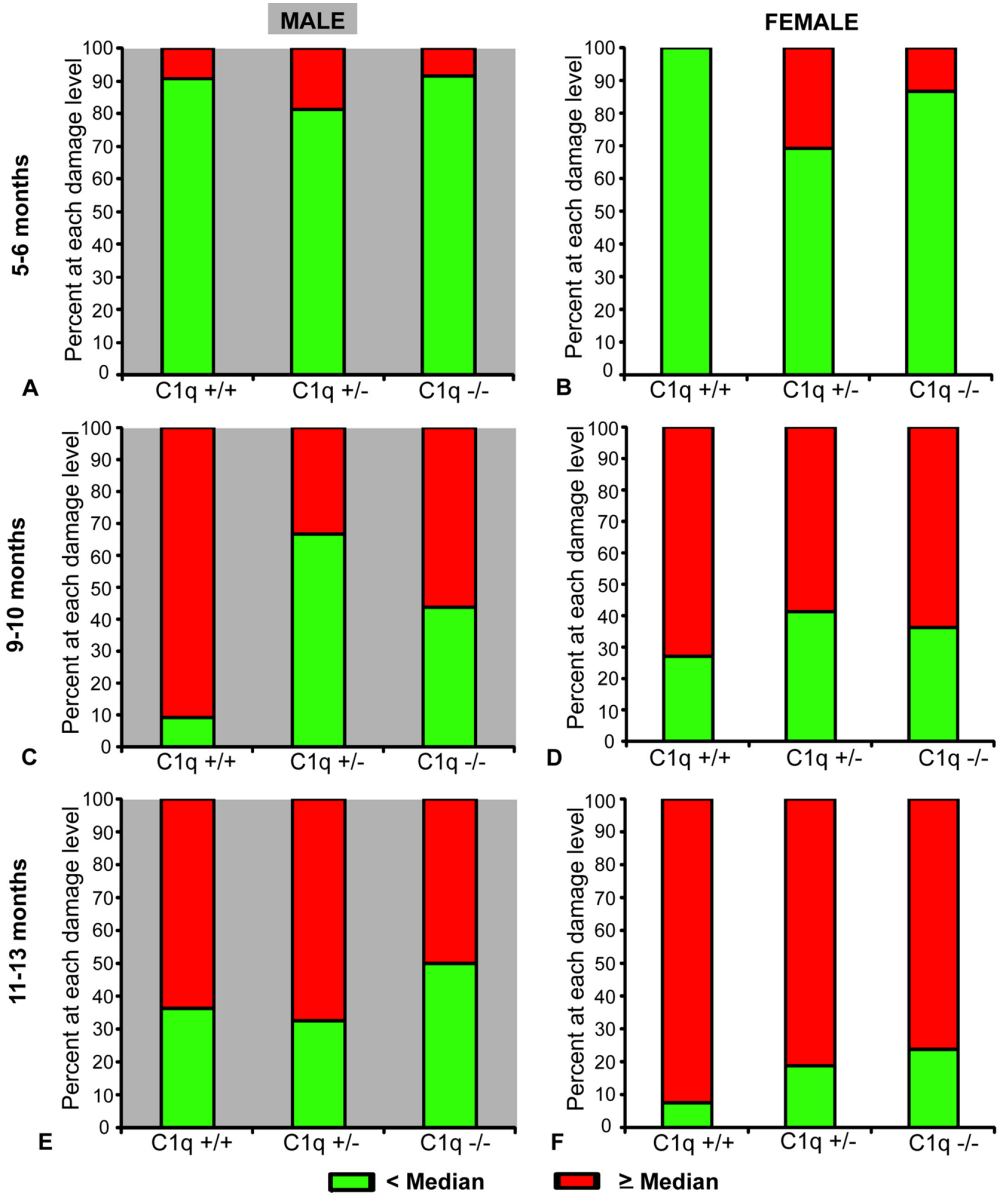
Proportion of eyes from male (A, C, E) and female (B, D, F) congenic C1qa DBA/2 mice with various levels of ON axonal degeneration at 5–6 (A, B), 9–10 (C, D) and 11–13 (E, F) months of age. Eyes are grouped as having ON axonal degeneration below (scores <3) or above (scores ≥3) the median.

Optic nerves were well preserved at 5–6 months of age in both male and female animals of all three genotypes (*p>0*.*4* and *p>0*.*7* for male and female mice respectively, ANCOVA, Fig 5A and 5B). In 9–10 month old male mice, mean ON scores were significantly lower in C1qa -/- and C1qa +/- animals compared to C1qa +/+ animals (*p<0*.*05*, post-hoc Fisher's LSD, *p<0*.*05*, ANCOVA, Fig 5A). In addition, the proportion of eyes that had ON scores below the median score (score of 3) was significantly higher among C1qa -/- mice compared to that in C1qa +/+ mice (*p<0*.*043*, Fisher exact test) (Fig 6C). At 11–13 months of age in animals of the male sex there was no significant difference in mean ON scores between the three genotypes (*p>0*.*5*, ANCOVA, Fig 5A). The proportion of eyes that had ON scores above or below median was also similar among the three genotypes (*p>0*.*44*, Fisher exact test, Fig 6E).

At 9–10 months and 11–13 months of age, there were no statistical difference in mean ON scores between C1qa +/+, C1qa +/- and C1qa -/- female mice (*p>0*.*7* and *p>0*.*07* respectively, ANCOVA, Fig 5B). Similarly, the proportion of eyes from female animals that had ON scores above and below the median were not significantly different between the three genotypes in the 9–10 and 11–13 month age groups (*p>0*.*75*, and *p>0*.*25*, Fisher exact test for animals 9–10 and 11–13 months of age respectively) (Fig 6D and 6F).

### C1qa absence alters microglia morphology prior to the onset of RGC or axonal loss in males

**Fig 7:**
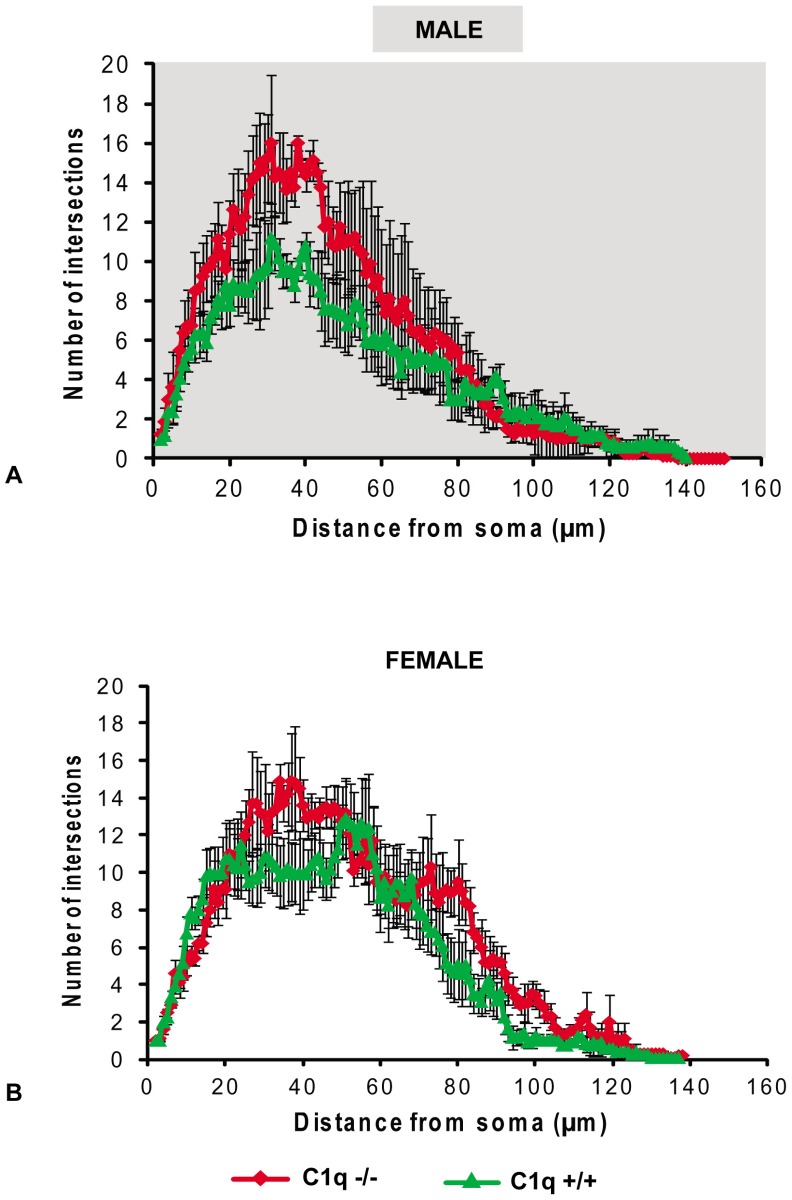
Mean (± SEM) number of intersections at various radii from the cell soma of retinal microglia located in the nerve fiber layer of eyes from male (A) and female (B) C1qa +/+ and C1qa -/- mice 6 months of age. Microglia images were obtained at a distance of 1000um from the optic nerve (2 per retina).

At 5–6 months of age, microglia in eyes of male C1qa -/- animals (n = 6 eyes) had significantly higher number of branches compared to those in eyes of C1qa +/+ mice (n = 4 eyes) (*p<0*.*004* and *p<0*.*03* respectively for average and maximum number of intersections, t-tests, Fig 7A). In contrast, there was no statistically significant difference in the average or maximum number of intersections between eyes of C1qa -/- (n = 5) and C1qa +/+ (n = 6) female animals of the same age (*p>0*.*25* and *p>0*.*52* respectively, t-tests, Fig 7B).

## Discussion

Our understanding of the role of complement in glaucoma is in large part derived from studies in DBA/2 mice [17, 21, 27]. Although, upregulation of various complement components occurs in various animal models of the disease [17, 28] as well as in humans with glaucoma [17], genetic manipulations in DBA/2 mice has allowed us a better understanding of how complement may be affecting the retina and optic nerve. However the picture is still incomplete.

In the present study, we used the DBA/2NNia mice that we maintain at SUNY Downstate to determine in detail the effects of C1qa ablation on RGC and axonal loss and attempt to understand the cellular mechanisms underlying these effects. The DBA2/NNia substrain of mice is closely related to the DBA/2J substrain widely used by many investigators. The animals used in the current study were derived from animals originally obtained from Dr. Sheldon in the early 90’s [29]. The animals underwent re-derivation in the mid-2000’s to generate a pathogen-free colony that has been since maintained in a barrier facility. We have previously reported on the pattern and amount of RGC loss of these animals [25] which appear to parallel those reported for the DBA/2J substrain by other investigators (albeit at a slightly later age) [18]. These animals also develop far less corneal pathology (previously reported for DBA/2J mice) which makes non-invasive IOP measurements more reliable. As with DBA/2J mice [19], DBA/2NNia mice carry mutations in the *Gpnmb* and *Tyrp1* genes which are responsible for the pathology. IOP starts to increase at approximately 6 months of age and remains elevated until the 12th month of age causing progressive RGC and axonal loss. Female mice develop damage slightly earlier than males.

We generated congenic animals by backcrossing C1qa KOs into the DBA/2 background. We backcrossed for 14 generations to ensure that the animals studied were in the DBA/2 background. Our effort occurred in parallel to and independent of a similar effort reported by Dr. John’s group in a 2012 paper [21]. We report our results here as a confirmation of the main point of that work. However, we provide significantly more detail in this report, including results in heterozygous animals, and highlight some significant differences between the current results and those reported by Howell et al [21].

IOP elevation was detected in both male and female animals after the 6^th^ month of age and remained elevated until the 12^th^ month of age in most of the animals. The IOP profile observed in C1qa +/+ animals is largely similar to what has been previously reported for DBA/2NNia mice which demonstrate IOP elevation slightly earlier compared to DBA/2J animals [30]. The IOP profiles of male and female mice were different: even though in males there was no significant difference in average IOP among the three genotypes, female animals with either haplo-insufficiency or complete deletion of C1qa had significantly higher IOPs. The reason for this difference in IOP response in female animals is unclear and contrary to what was previously reported [21]. However it does suggest that complement may participate in IOP regulation. In fact a number of studies have detected various complement components in the aqueous humor [31–35]. Furthermore, DBA/2 animals have mild anterior chamber inflammation that contributes to anterior chamber synechial formation [18, 19]. Perturbation of complement activation may affect development of angle closure in these mice and thus lead to higher IOP. The presence of a sex difference suggests that hormonal or other factors may modulate this response.

As expected, no significant RGC or axonal loss was present in all three genotypes studied at 6 months of age. Because of this obvious lack of difference in appearance of both retinas and optic nerves we focused our quantitative (and more time consuming) analysis on the potential differences at later time points. As expected, both male and female C1qa +/+ animals lost RGCs with increasing age. At least 60% of the eyes from C1qa +/+ animals studied at 9 and 12 months of age had less than 25,000 remaining RGCs (retinal scores of 0–4). Female C1qa +/+ animals had an average of ~23,000 RGCs per retina at 9 months of age which declined further to ~10,000 RGCs per retina at 12 months of age. In male C1qa +/+ mice, RGC loss was slightly less severe with ~19,000 RGCs remaining at 12 months of age. Similarly, C1qa +/+ animals (both male and female) exhibited significant axonal degeneration at both 9 and 12 months of age.

C1qa deletion resulted in enhanced RGCs survival for males at 9 (and only marginally at 12 months of age) and for females at 12 months of age. The increased average number of RGCs in C1qa -/- animals compared with the average RGC numbers in C1qa +/+ animals was also reflected in an increase in the proportion of animals with mild RGC loss (RGC scores 8.5–10). Eyes of C1qa +/- animals appeared to have less damage than those of C1qa +/+ mice.

Optic nerve axonal degeneration was similar between the three genotypes in female animals of all age groups and in males at 11–13 months of age. Only at 9–10 months of age C1qa +/- or C1qa -/- males enjoy any protection compared with C1qa +/+ animals.

The protections afforded to C1qa +/- animals is an intriguing finding. Although one cannot exclude the possibility that the observed differences may arise from a relatively small sample size (from 6 to 32 for ONs and from 16 to 34 for RGCs for each sex and group) due to high heterogeneity and asynchronicity of damage inherent to the DBA/2 glaucoma model, haploinsufficiency of C1qa may lead to lower levels of C1q and consequently less complement activation. The different effect of C1qa haploinsufficiency on RGC versus axonal survival suggests that complement may have different functions in the retina and optic nerve as additional data from C3 knockout animals suggests (Howell G, Rudin Glaucoma Prize 2013 lecture).

The above results are interesting in view of the results previously reported by Howell et al [21] on the effect of C1qa deletion on neurodegeneration in DBA/2 mice. In that paper the authors described significant protection afforded to C1qa -/- DBA/2 mice at both 10.5 and 12 months of age. Protection was detected in both ON axons and RGC somata. However, IOPs in those experiments were similar in C1qa -/- and C1qa +/+ animals. Although not clearly identified in the methods section of that paper, the animals used were of a mixed sex population (G Howell–personal communication).

It is important to understand that there are significant methodological differences in the work reported by Howell et al [21] and the current work.

First of all, the ON scoring system employed by Howell et al [21] is not linear across the whole spectrum of glaucomatous optic nerve degeneration. Optic nerves in the “Severe” group in that report had 50% or more axonal loss (equivalent to scores of 3–5 in the current cohort), while animals in the “Mild to no damage” group had less than 30% axonal loss (equivalent to scores of 1–2.2 in the current cohort). The non-linearity of that classification system is useful for separating out the eyes with very early disease, but it does not accurately quantitate the amount of damage in eyes with more significant axonal loss. In comparison to Howell et al method, the scoring method employed in the current report divides the continuum of all possible damage levels into equal intervals which allows for linear scoring of various degrees of damage. This allows more optimal comparisons of eyes showing extensive damage like the ones encountered in this dataset.

RGC quantification is similarly performed in a completely different way in the Howell study [21] compared with the current one. In the previously published work [21], RGC quantification is performed by histological staining and imaging of a very small area (~2%) in the inner retina. However this method cannot determine whether the RGCs present in the inner retina are connected to their CNS target. In the present work, we quantified RGCs throughout the retina by retrogradely labeling them with fluorogold. By quantifying 100% of the RGCs we overcome the well-known problem of focal (or patchy) RGC loss that is associated with DBA/2 glaucoma [25, 36]. Even more importantly, labeling of RGCs with fluorogold proves that these cells are still connected to the superior colliculus (the primary target for RGCs in mice) and still have intact axonal transport and thus are potentially salvageable even if damaged.

Other potential differences that may influence the results in the two studies include: strain differences between DBA/2J and DBA/2NNia mice caused by possible genetic drift between these strains; congenic interval differences from backcrossing the C1qa mutation (in the current study we backcrossed mice for 14 generations whereas in the Howell et al study the mice were backcrossed for 8 generations); unavoidable environmental differences related to mouse

**Fig 8:**
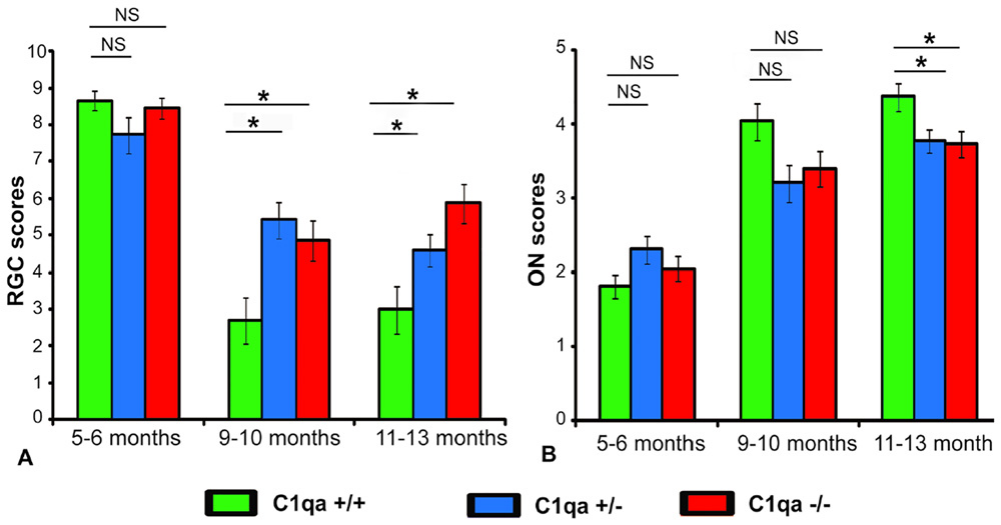
Semi-quantitative RGC scores (A) and ON scores (B) of all the animals (both male and female) analyzed. Each point represents the mean (±SEM) of between 22 and 55 eyes. C1qa +/+ animals are statistically significantly different from other groups at 9–10 months of age for RGC scores only (p<0.005, ANCOVA, Fisher LSD post-hoc comparisons) and for both RGC and ON scores at 11–13 months of age (p<0.005 and p<0.03 respectively, ANCOVA, Fisher LSD post hoc comparisons). Statistically significant differences in post-hoc comparisons are indicated by a (*). NS: no statistical significance.

To provide a more direct means of comparing the two studies, we reanalyzed the data presented in the Results section above after combining datasets from both sexes. These mixed sex results are presented in Fig 8. As can be seen, C1qa +/+ animals (mixed sex) show significantly higher amounts of RGC loss at both 9–10 and 11–13 months of age, while they show significantly higher amounts of axonal loss only at 11–13 months of age. It should be noted that after combining datasets of male and female mice, sample sizes are of similar magnitude to the ones reported by Howell et al.

Thus, despite the significant differences in methodology between the two studies, the results from the current one confirm the conclusion reached by Howell et all that C1qa absence is protective for both RGCs and their axons at least during some time in the course of the disease. The level of protection differs between the two studies and this difference is probably related to the difference in IOP exposure in the two cohorts.

It appears though that C1qa absence eventually overcomes the effect of higher IOP on RGCs in C1qa -/- female mice. It also suggests that C1qa upregulation early in glaucoma probably acts independently of high IOP to promote RGC loss. In fact, we have previously shown that short-term IOP increase does not cause C1qa upregulation in the retina [37], making the cause of C1qa upregulation in glaucoma unclear.

The difference in IOP (with higher IOPs in C1qa -/- compared to C1qa +/+ female animals in the current study) suggests that axonal loss is more sensitive than RGC loss to the effects of IOP and less sensitive to the presence or absence of C1qa.

The results of the current study in male animals, where IOP was similar between the three genotypes, also partially confirm the above conclusions. RGCs were protected by C1qa absence at 9–10 months of age suggesting that C1qa participates in the damage of RGCs. C1qa absence is also axoprotective in male animals at 9–10 months of age. However, both the RGC protection and the axoprotection seem to be lost by 11–13 months of age (despite a slight difference in RGC protection at this age group manifesting as a smaller proportion of retinas with extreme RGC loss) suggesting that C1qa absence cannot fully abrogate glaucomatous degeneration ad infinitum. The differential effectiveness of C1qa absence in overcoming IOP induced RGC and axonal loss probably reflects the fact that these are processes that are mediated separately.

**Fig 9:**
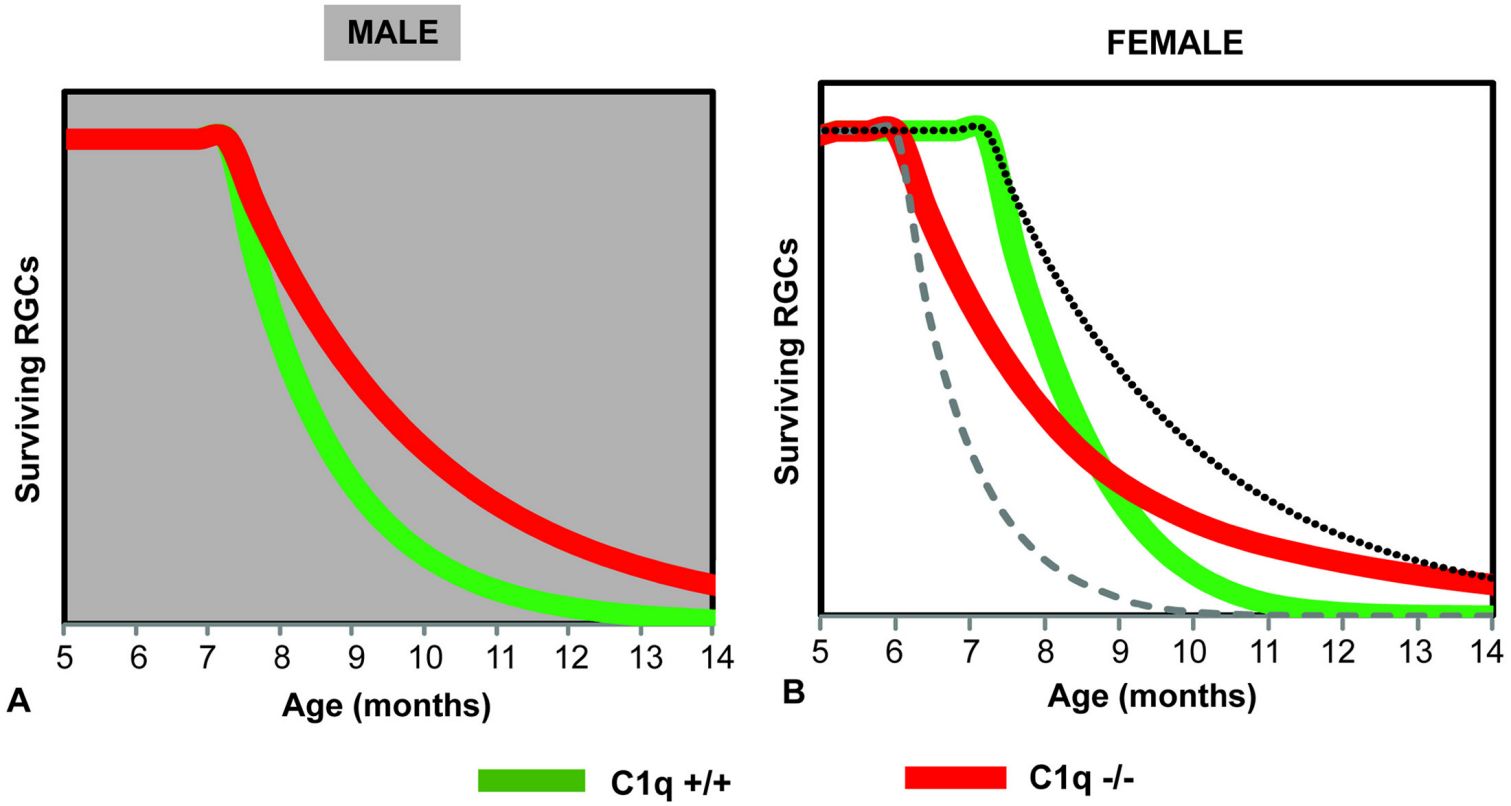
Modeling of RGC loss in C1qa -/- and C1qa +/+ congenic male (A) and female (B) mouse eyes. Absence of C1qa decreases the rate of RGC loss in male animals, resulting in a significant difference at 9–10 months of age and a smaller difference later in the course of the disease. A similar effect is not observed in females as increased IOP in C1qa -/- animals shifts RGC loss to earlier time-points, making RGC differences only detectable at 11–13 months of age. Dashed line indicates the theoretically expected RGC loss in C1qa +/+ mouse eyes if they had IOP elevation similar to the levels observed in C1qa -/- mice. Dotted line indicates the theoretically expected RGC loss in C1qa -/- mouse eyes if they had IOPs similar to their C1qa +/+ littermates.

To better illustrate the role of C1qa on RGC loss we generated a model (Fig 9 that is based on the data presented above. According to the model, RGC loss in male animals (Fig 9A) proceeds in an increasingly accelerating fashion between 8 and 12 months of age. C1qa absence slows the rate of RGC loss resulting in a larger RGC difference around 9 months of age and a smaller one closer to 12 months of age. In female animals (Fig 9B), the picture becomes more complicated by the different IOP exposure of C1qa -/- and C1qa +/+ animals. IOP would be expected to shift the RGC loss curve to the left (dashed line in Fig 9B), while C1qa absence would be expected to slow the rate of RGC loss as in the male animals (dotted line in Fig 9B). C1qa -/- animals would be affected by both of these opposing influences, thus resulting in a curve that while it shows no difference from C1qa +/+ mice in the amount of RGC loss at ~9 months of age, exhibits some protection at ~12 months of age.

To further understand how C1qa absence may be mediating its RGC protective effects we examined retinal microglia at 5–6 months of age. We and others [3, 6, 21, 38, 39] have previously shown that retinal microglial activation is important in glaucoma and that it can be detected by observation of morphological changes as microglia becomes activated. We reasoned that comparing the microglial activation status of C1qa -/- and C1qa +/+ mice at 5–6 months of age (a time when no significant RGC (or axonal) loss has occurred) we could determine whether the effect of C1qa on the glaucomatous pathology was mediated via microglial activation. Interestingly, C1qa +/+ male mice had significantly more activated microglia (as detected by a number of morphological parameters) than their C1qa -/- littermates at 5–6 months of age suggesting that C1qa presence enhances such activation. It is interesting to note that RGC and axonal survival is better in male C1qa -/- mice at 9–10 months of age. It is unclear why retinal microglia are less (and equally) active in both C1qa +/+ and C1qa -/- female animals at 5–6 months of age while a difference exists in male mice of the same age. It is possible that this difference is related to the role of estrogens present in younger female animals. Estrogens are known to affect the levels of microglial activation and may counteract the presence of elevated C1qa [40–42].

In summary, we present a detailed characterization of the effects of C1qa loss on RGC and axonal loss in a mouse model of glaucoma. Our results generally support the conclusions of a prior report [21] that C1qa absence is protective for RGCs. It appears that the effect is eventually lost as the disease continues to progress. In addition, axoprotection afforded by C1qa absence is also transient and is overcome by exposure to higher IOPs. Evidence from examination of the state of microglial activation supports an effect of estrogens in modulating the effect of C1qa presence.
